# Implementation of a three-pillar training intervention to improve maternal and neonatal healthcare in the Democratic Republic Of Congo: a process evaluation study in an urban health zone

**DOI:** 10.1080/16549716.2021.2019391

**Published:** 2022-01-10

**Authors:** Marie Berg, Sylvie Nabintu Mwambali, Malin Bogren

**Affiliations:** a Institute of Health and Care Sciences, Sahlgrenska Academy, University of Gothenburg, Gothenburg, Sweden; b Department of Obstetrics and Gynecology, Faculty of Medicine and Community Health, Evangelical University of Africa, Bukavu, Democratic Republic of Congo

**Keywords:** Healthcare providers, maternal and newborn health, Democratic Republic of Congo, low-income countries, implementation research

## Abstract

**Background:**

Numerous quality-improvement projects including healthcare professional training are conducted globally every year, but there is a gap between the knowledge obtained in the training and its implementation in practice and policy. A quality-improvement programme was conducted in eastern Democratic Republic of Congo (DRC) to reduce maternal and neonatal mortality and morbidity.

**Objective:**

This study explores the implementation process, mechanisms of impact, and outcomes of a training intervention addressing labour and birth management and involving healthcare providers in an urban health zone in eastern part of DRC.

**Methods:**

In 2019, master trainers were educated and in turn trained facilitators from seven participating healthcare facilities, which received the necessary equipment. Data comprised statistics on maternal and neonatal birth outcomes for the years before and after the training intervention, and focus group discussions (n = 18); and interviews (n = 2) with healthcare professionals, at the end of (n = 52) and after the training intervention (n = 59), respectively. The analysis was guided by a process evaluation framework, using descriptive statistics and content analysis.

**Results:**

The three-pillar training intervention using a low-dose, high-frequency approach was successfully implemented in terms of fidelity, dose, adaptation, and reach. Several improved care routines were established, including improved planning, teamwork, and self-reflection skills, as well as improved awareness of the influence of the care environment, of having a respectful encounter, and of allowing a companion to be present with the birthing woman. The proportions of emergency caesareans decreased and of vaginal births increased without an increase in maternal and neonatal complications.

**Conclusion:**

The findings of this study are encouraging and provide learnings for other healthcare facilities in DRC as well as other low-income countries. When designing similar training interventions, it is crucial to consider contextual factors such as incentives and to measure more salutogenic outcomes.

## Background

The period around childbirth is critical to saving the lives of women and newborns [1,2], and poor-quality care is considered a greater barrier to reducing mortality and morbidity than insufficient access to care [3,4]. Providing high-quality care is thus critical to achieving the Sustainable Development Goals’ (SDGs’), and especially goal three on health with the target aim to reduce the global maternal mortality and end preventable deaths of newborns and children [4]. Fundamental components of good-quality care at facility-based healthcare services include care that is safe, effective, timely, efficient, equitable, and person-centred [1].

Poor or insufficient care quality remains a global challenge, especially in low-resource settings such as the Democratic Republic of Congo (DRC), which, according to the latest national statistics (as of 2013), had a maternal mortality rate of 846 per 100,000 live births and a neonatal mortality rate of 28 per 1000 live births [5]. Although these statistics are old, there are indications that maternal and neonatal mortality and morbidity remains too high [6], despite 80% of mothers giving birth at healthcare facilities as of 2013 [5].

A main component of good-quality maternal and newborn care is that healthcare providers have the right competencies in maternal and neonatal care [2]. In-service training has long been used to improve such competencies, although with varying degrees of success [7]. Repetitive training interventions can, according to a systematic integrative review, result in better learning outcomes and could sustain the learned skills, transferring them to clinical performance. However, research from low- and middle-income countries is limited [8]. Systematic reviews have reported on the impact of competency-based training used in low-income settings on preventing and managing labour-, birth-, and neonatal-related complications [9,10]; however, to our knowledge, no available evaluations target the full implementation process and the mechanisms of impact of the training intervention.

Despite the billions of dollars spent globally each year on quality-improvement projects such as healthcare professional training, there is a gap between the knowledge obtained in the training and its implementation in practice and policy. To obtain tools to reduce this gap, there is need for implementation research that includes active and conscious studies of the processes of integrating new ways of working into daily practice [11].

As part of a larger quality-improvement programme in the DRC intended to reduce maternal and neonatal mortality and morbidity, we have explored the implementation process, mechanisms of impact, and outcomes of a training intervention offered to healthcare providers. The lessons learned from the results are thought to be useful in the DRC and other low-income countries when designing and implementing similar training interventions.

## Methods

### Study design and ethical approval

The study, approved by the National Ethical Committee of Public Health (CNES 001/DPSKI/129PM/2019), used an exploratory design guided by the principles of an evaluation framework developed by Moore et al. [12] that gives freedom in using the framework components. The context and its influence on the training intervention are described elsewhere (manuscript submitted), while this study describes the implementation process, quality improvements, and mechanisms of impact. The research questions are presented in detail in Table 1.

**Table 1.** Components of the process evaluation of the three-pillar training intervention

Implementation process
Fidelity: Was the training delivered as intended? Were any adaptations or alterations made in order to achieve better contextual fit? If yes, what were they and when were they made? What did the training do well? How and why?
Dose/Exposure To what extent were the training milestones and targets achieved? To what extent has the training resulted in change? Why?
Reach To what extent were the healthcare providers involved in the training? How could the healthcare providers’ involvement have been improved?
Acceptability Was the training programme acceptable and perceived as relevant? Was the training programme useful, and what challenges were encountered? How could the training programme have been improved?
Improvements and mechanisms of impact
How did the training programme affect healthcare providers’ care routines, and what were the mechanisms of impact? Were there any improvements in maternal and neonatal health outcomes?

### Setting

The DRC comprises 26 provinces, with more than 500 health zones that deliver healthcare at three levels of facilities: healthcare centres, district (secondary) hospitals with the capacity to perform C-sections, and referral hospitals (one per health zone) [13]. The healthcare sector is characterized by public underfunding and insufficient infrastructure, and the functioning of health facilities is essentially ensured by patients’ payments [6]. The healthcare facilities are governed by either the governmental or private sector, but most are private or run by confessional communities due to lack of government capacity.

The study was conducted in a health zone situated in the provincial capital of South Kivu Province, eastern DRC, with 40 healthcare facilities comprising 34 healthcare centres, five district hospitals, and one referral hospital. In 2018, before the intervention, there were 450,000 residents and 16,101 registered births in this health zone.

### Training intervention

The training intervention was conducted in 2019. We developed a three-pillar training intervention based the causal principles of person-centred holistic care [14]. Pillar 1 focused on theory and activities to promote normal physiological births and was based on a model of woman-centred childbirth care [15], using equipment from Laerdal Global Health [16], a birthing ball and a ‘Rebozo technique’ sheet [17]. Pillar 2 focused on preventing and managing complications and followed the content and pedagogy of the programmes Helping Mothers Survive Bleeding after Birth (HMS-BAB) and Helping Babies Breathe (HBB) [18], the Laerdal products MamaNatalie Complete, MamaBirthie, and NeoNatalie Complete [16]. The third pillar consisted of group reflection following a process-oriented group reflection model focusing on healthcare professionals’ self-reflection skills and self-confidence [19].

The hypothesis was that the three-pillar training intervention, using low-dose, high-frequency training pedagogy, would improve the quality of care, resulting in more spontaneous vaginal births and fewer caesarean sections and ultimately leading to reduced maternal and neonatal mortality and morbidity.

The low-dose, high-frequency pedagogy is defined as short, targeted in-service simulation-based learning activities, which are spaced over time and reinforced with structured, ongoing practice sessions on the jobsite [20].

### Implementation strategy

The implementation of the training intervention was steered by a multi-professional project committee of healthcare professionals from the DRC (*n* = 3) and Sweden (*n* = 3), including the authors of this paper (i.e. MBe, SNM and MBo).

The Congolese committee members chose and invited seven of the 40 healthcare facilities to be targets of the training interventions: three healthcare centres (ID numbers 4, 6, and 7), three district hospitals (ID numbers 2, 3, and 6), and the referral hospital (ID number 1). All were privately governed, reflecting the fact that there are almost no government healthcare facilities in the chosen health zone. All seven healthcare facilities accepted to participate. There were 7667 registered births at these seven healthcare facilities the year before the intervention.

**Figure 1.**
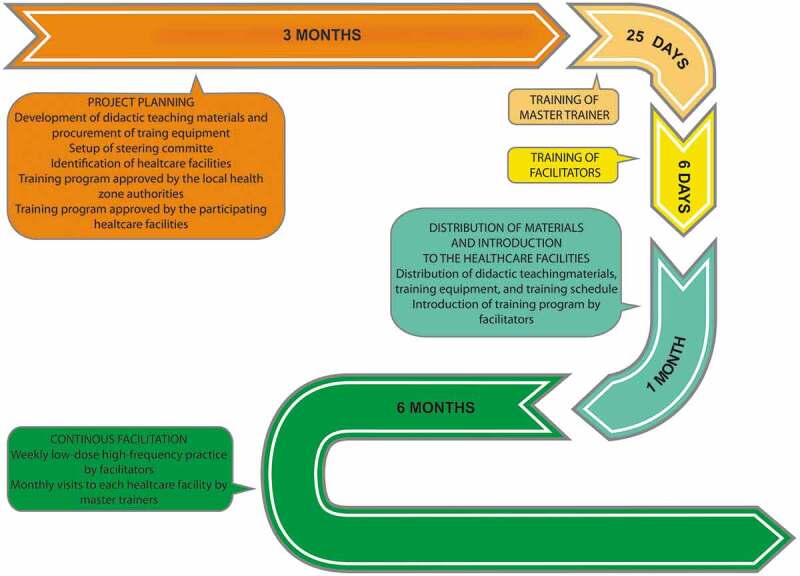
Roadmap on the implementation process of the three-pillar training program

Figure 1 presents details of the three-pillar training intervention. The project committee selected four healthcare professionals at the referral hospital to become master trainers: three nurses working as midwives and one physician specialising in gynaecology. They received 25 days of training in the three-pillar programme. Next, the facilities selected healthcare professionals to function as facilitators: two each from six of the healthcare facilities, and one from the smallest one. Ten were nurses or midwives and three were doctors, of whom two were gynaecologists and one a paediatrician. These 13 selected facilitators received six days of training given by the master trainers, supported by specialists in the respective pillars. After training completion, the seven healthcare facilities each received the following equipment together with teaching material on the three pillars: a birthing ball, a sheet for applying the Rebozo technique, and the Laerdal products MamaNatalie Complete, MamaBirthie, and NeoNatalie Complete.

A training schedule of weekly short training activities for a six-month period was defined in collaboration between the project committee and master trainers. This included weekly training in pillars 1 and 2 supported by the chosen facilitators at respective healthcare facility, and process-oriented group reflection once per month. During the six months, the master trainers continuously mentored the facilitators and conducted monthly follow-ups at the healthcare facilities during which they also led the process-oriented reflections with the staff. The master trainers received a small monetary incentive for their work, while the facilitators did not receive any monetary incentive.

### Data collection

Both qualitative and quantitative data were collected. Managers at each participating healthcare facility were informed about the study and approved it.

Qualitative data were collected on two occasions (i.e. at the end of training intervention and 14 months afterwards) at each facility through focus group discussions (FGDs) and individual interviews. All available healthcare staff working at the maternity units were invited to participate. All invitees were given verbal and written information about the study, including the fact that participation was voluntary and that they had the right to withdraw at any time without explanation.

Nine FGDs with a total of 52 participants were conducted on the first occasion, and nine FGDs and two individual interviews with a total of 59 participants were conducted on the second occasion. The characteristics of the study participants are described in Table 2. FGDs were conducted by MBe and MBo on the first occasion and FGDs and individual interviews by MBe on the second occasion. The FGDs were held in French by MBe, and on the first occasion were translated simultaneously into Swedish to MBo, who made field notes and asked clarifying questions. All FGDs and the individual interviews were audio-recorded and lasted 30–60 minutes (mean 45 minutes).

**Table 2.** Characteristics of study participants at each period

Variable	Period 1 (*n* = 52)	Period 2 (*n* = 59)
Sex		
Female	41 (79%)	47 (80%)
Male	11 (21%)	12 (20%)
Age range, years (mean)	23–71 (41.5)	23–72 (44.2)
Profession		
No formal health profession	5	8
Midwife	22	17
Nurse	14	24
Physician	11	10

Descriptive statistics on maternal and neonatal birth outcomes were collected directly from each of the participating healthcare facilities for the year before (2018) and after (2020) the training intervention.

### Data analysis

The basic principles of deductive content analysis [21] were applied, using the chosen components (Table 1) of the evaluation framework to guide [12] the analysis of the FGDs and interviews. First, all transcripts were read several times. Next, in new readings, meaning units were identified that answered the research questions. The meaning units were then compared and sorted based on similar content, and were further compared and clustered according to the research questions. The analytical process was completed by MBo and MBe separately, with repeated discussions until full agreement was reached. The third author followed this analysis process and approved on the final version.

The statistical analyses were performed with SAS Software, version 9.4. For comparison between two time periods, Fisher’s exact test was used for dichotomous variables and the Chi-square test for non-ordered categorical variables. Differences between groups were calculated in percent with 95% confidence intervals. All the tests were two-sided and conducted at the 5% significance level. As the training intervention was assumed to influence the percentage of vaginal births and emergency sections, and not planned caesareans, in Table 3 we first present descriptive statistics on all types of births, and then an analysis of vaginal births versus caesarean births.

**Table 3.** Comparison of maternal outcomes all healthcare facilities before and after the training intervention

Variable	2018 (n = 7525)	2020 (n = 7152)	p-value	Difference between groups Mean (95% CI)
Mode of birth				
Vaginal non-instrumental	5888 (78.2%)	5747 (80.4%)		
Vaginal instrumental	4 (0.1%)	6 (0.1%)		
Planned caesarean	570 (7.6%)	541 (7.6%)		
Emergency caesarean	1063 (14.1%)	856 (12.0%)	0.0014	
Mode of birth				
Vaginal, non-instrumental and instrumental	5892 (84.7%)	5753 (87.0%)		−2.3 (−3.5; −1.1)
Emergency caesarean	1063 (15.3%)	856 (13.0%)	0.0001	2.3 (1.1; 3.5)
Prophylactic uterotonic given within 1 min of birth*				
No	38 (0.6%)	3 (0.1%)		0.6 (0.4; 0.8)
Yes	5854 (99.4%)	5750 (99.9%)	0.0001	−0.6 (−0.8; −0.4)
Postpartum blood loss >500 ml				
No	7436 (98.8%)	7053 (98.6%)		0.2 (−0.2; 0.6)
Yes	89 (1.2%)	99 (1.4%)	0.31	−0.2 (−0.6; 0.2)
Retained placenta >30 min after birth				
No	7498 (99.6%)	7110 (99.4%)		0.2 (−0.0; 0.5)
Yes	27 (0.4%)	42 (0.6%)	0.057	−0.2 (−0.5; 0.0)
Maternal death				
No	7509 (99.8%)	7145 (99.9%)		−0.1 (−0.3; 0.0)
Yes	16 (0.2%)	7 (0. 1%)	0.12	0.1 (−0.0; 0.3)

For categorical variables n (%) is presented. For comparison between groups Fisher´s Exact test (lowest 1-sided p-value multiplied by 2) was used for dichotomous variables and Chi Square test was used for non-ordered categorical variables. The confidence interval for dichotomous variables is the unconditional exact confidence limits. If no exact limits can be computed the asymptotic Wald confidence limits with continuity correction are calculated instead.

*This variable is based on total number of vaginal births.

## Results

In presenting the results, the FGDs conducted in the two periods are labelled with the FGD number followed by a (first period) or b (second period), respectively, with the facilities where they were held labelled 1–7, as described in the section ‘Implementation strategy’.

### The implementation process

#### Fidelity, dose, exposure, and reach

Overall, the training programme was delivered as planned and considered easy to follow. It fitted the contextual situation, and no revisions had to be made in the manual. The principles of conducting low-dose, high-frequency training functioned well, and the weekly schedule facilitated prioritisation, with some variation between the participating facilities. Training activities relating to pillars 1 and 2 were conducted once or twice a week with a few up to 32 staff members present on the occasions; in addition, some facilities also conducted training spontaneously, initiated by the staff themselves. The training sessions lasted 15–60 minutes. Pillar 3 – the group reflection – was conduced as 1.5-hour sessions once a month and led by the master trainer responsible for each healthcare facility. All staff members were actively involved, although most physicians did not participate as much as expected:

We train twice a week. It can be pillars 1 or 2 or both. We use the mannequins and the Rebozo sheet. The group reflection sessions are led in Swahili. We have found a method that makes us stop and reflect. (6a)

#### Acceptability

*Usefulness*: The three-pillar training programme, comprising specific materials and training techniques combined with systematic reflection, was considered very useful. The programme covered how to behave towards the birthing woman and her companions, the use of alternative techniques, and better planning. It was repeatedly mentioned that the training programme provided skills that promoted physiologically normal labour and birth, preventing and managing complications. The group reflection sessions of pillar 3, a completely new activity, were considered very useful. These sessions improved the teamwork skills of participants without adopting an accusatory dynamic. Previously, the care providers had been fearful and reluctant to talk about what had gone wrong and to ask for help; in contrast, the structured and secure group reflection sessions made them feel safe and calm:

Everything has changed. I am very encouraged by the training and have improved my skills. We have gained new knowledge, for example, in using the Rebozo technique and massaging the back. Everything in the training has been useful. (7b)

Since group reflection started, we have learned about our mistakes and from our experience. In groups, everyone gave their own experience based on their work. Then, based on this, we identified strengths and weaknesses …. I think that the most useful thing is to become aware, increased awareness of the whole team to apply what you have learned. That it is integrated into everyone’s bodies helps us to do a good job in practice. (9b)

*Challenges*: Although the training programme was experienced as very positive overall, some challenges were encountered. The lack of incentives was one such challenge, and the minimum should be reimbursement for time and travel to and from work when participating in one’s spare time. The scheduled practice sessions in pillars 1 and 2 were often conducted in the mornings, which made it challenging for those who had been working the night shifts.

Doctors did not participate as much as the other staff did, although they supported the programme. At one hospital, the doctors refused to participate even during working hours, as they did experience ‘ownership’ of the programme through being paid to participate:

It was difficult to convince the staff to participate in the various training sessions, as there were no financial incentives to offer those who came during their leisure time. Sometimes they would refuse to come because they did not get paid for the transport. It was also difficult to motivate the staff to stay for training after the end of the working day. (8)

The didactic materials and equipment provided for training and practice were mentioned as a great strength of the training programme. However, every facility lacked space in which to unpack the material and keep it safe from being stolen:

All training material is packed in the bag and must be picked up for each training occasion. This is due to lack of space. (5a)

A need for refresher training was stressed in the follow-up interviews 14 months after training programme completion. The rationale was that this would keep the important new knowledge fresh and updated in the care routines. There was also a desire to increase the number of facilitators.

This training is good, but to make a recommendation, you should continue and give the training regularly, for example, every three months, and you could then give this training here at our centre. Then many more could participate. (3b)

#### Improved care routines and associated mechanisms of impact

The clinical usefulness of the training programme was obvious, and was mentioned by care providers at the healthcare facilities, by the master trainers and by the local project leader: ‘The three pillars have really helped us to improve our ways of working’ (8).

#### Planning, teamwork, and reflection

The three-pillar training had improved care planning and the awareness that preparedness for complications prepared the staff to act immediately when there was a need. Furthermore, the training influenced awareness of the importance of working in an interdisciplinary way, which enhanced patient safety:

I have become aware of the importance of being prepared well in advance. Having forethought calms us down and, as we are active all the time, this reduces fatigue. Before, we ran all the time, were stressed, and tried to get the material together only when it was time for birthing. (1b)

Since the training, I have become aware that it is important to work in teams. Those who have followed the model with the three pillars work more and better in teams with different professions, such as nurses, midwives, doctors, and lab staff. (10)

The process-oriented group reflections created an awareness of the value of continuously reflecting on recently performed care. Reflecting on situations without judging helped prevent the repetition of mistakes and allowed learning from care situations that went well. Some facilities continued with the monthly reflection sessions after finalised training, while others started to conduct reflection sessions in daily practice, following the process-oriented group reflection principles:

We have continued with reflection groups, once a month. The doctor is responsible. We evaluate ourselves. Everyone is encouraged to say something, to tell a story. But we have not followed the method exactly as it was. (4b)

The reflection groups have changed us. Now we talk regularly about the handling of difficult, complicated cases. It is not a place to condemn each other without support. (6)

#### Respectful approach, involvement of companions, and an enabling care environment

The training raised the participants’ awareness of the importance of a respectful approach to the birthing woman and of involving her companion of choice, from reception at the facility to birth. This included following the woman’s wishes and *creating a loving atmosphere* (2a):

Before, we had no good communication with the mother. Now we welcome her in a different way that positively affects her labour. Then the woman feels at home. The women have noticed that we are different, they have commented on it. With good reception, the woman becomes calm. … Being close to the woman is always positive. It helps the woman to feel safe, which helps alleviate the aches and pains [that happen] if she’s scared, then the pain stops. We encourage the woman, clearly explain what will happen, everything we are doing and why, and the results of surveys. Now that we are close, we can continuously evaluate how the woman is feeling and the course [of the birth]. … Being close to the mothers has an effect, it results in more normal births. … The only disadvantage is that we do not have the strength or time when there are many women who need support. Especially at night it is difficult. (3b)

The training made the participants aware of the importance for the birthing woman of providing continuous supportive care, including having her husband or other companion close by and involved:

Before, the person who accompanied the woman was not allowed to be involved, but now we are collaborating with the woman’s companion. They may enter the birthing room if necessary. Collaborating with the companion is very helpful. (4b)

Furthermore, the training made the participants aware of the influence of the care environment on labour and birth. For example, they now understand that it is counterproductive to receive a woman and have the first talk with her in a corridor where it is impossible to safeguard her privacy, making it difficult get to know her and her history in order to develop an optimal individual care plan. Some facilities created better reception conditions, but overall, it was still difficult to find a place to be alone with the woman:

Before, we received the woman in a corridor – there the woman was not comfortable, and a lot of people came by, disturbing her. But now she is received in a quiet room, without others present. This gives her peace, and she can share her story. Confidentiality is thus improved, which promotes the oxytocin in the woman’s body, compared to when many people pass by, which is stressful. (1b)

#### Birth-enabling practices

Through the training programme, the participants learned about and put in place new practices to promote and enable uncomplicated physiological births. These practices included using alternative birthing positions, promoting the mother–child connection, and managing complications. There was awareness that the birth was more likely to be safe and healthy when unnecessary interventions that disrupted the normal physiological processes were minimized. This was experienced and observed by both the participants themselves and the doctor in charge.

The use of alternative birthing positions included encouraging the labouring woman to move and change positions and to push in a four-foot position. The techniques, such as providing massage and encouraging the women to move around and to use the birthing balls and Rebozo sheets, made the women feel in control and in need of less pharmacological pain relief. It also helped the foetus to descend and resulted in a more spontaneous birth, preventing medical interventions such as Caesarean sections:

Before, we did not even know about these methods, for example, if a woman had problems, they immediately decided on caesarean section and took her directly to surgery, but now we have tools for working with the woman and she often gives birth herself with the help of these tools. (2b)

The initial care of the newborn has improved in several ways due to the training. The newborn is now immediately placed skin-to-skin with the mother, and the umbilical cord is cut only when the pulsations in the umbilical cord have stopped. The education also explained why it is important to give prophylactic uterotonic after birth. According to the participants, this has resulted in improvements such as fewer transfers of the newborn to higher care levels, and hindered the companions to take the newborn and give traditional medicine:

It has contributed to better care for the mother and child. We have improved our routines. For example, before we dressed the baby immediately after the birth, [but now] we put the baby skin-to-skin. Previously, we did not wipe the baby to protect against the cold, which we do now. Before training, we did not know about the effects of oxytocin, such as the uterus contracting and reducing bleeding, but now we do. Now I see that colleagues give oxytocin routinely. We have contributed to counteracting PPH [i.e., postpartum haemorrhage]. We have really developed many good routines that promote optimal care. (8)

Several routines for managing complication have been put in place. A woman presenting with a life-threatening complication such as post-partum bleeding is now cared for more systematically through observation, assessment, diagnosis, and immediate action. Treatment of newborns with asphyxia has also improved, as this quotation shows

Three days ago I [i.e., a doctor] was called to a child who had Apgar 6 at birth. When I arrived they had started resuscitation, and I found that they were already using the assisted ventilation, and after five minutes, the baby started to breathe. I could see that the staff could handle the case correctly. When the child and mother left, we produced simulation material and then rehearsed resuscitation again. We called in others who work in childbirth and went through the case again. (4a)

#### Maternal and neonatal outcomes before and after the training intervention

This study design does not allow to show the direct effect of the training intervention. However, it is interesting to study the differences before and after the training intervention conducted July to December 2019) for maternal and neonatal outcomes. In this paper, we present statistics the year before (2018) and the year after (2020) for all facilities together. Details on differences between groups and confidence intervals are described in Tables 3 and 4.

**Table 4.** Comparison of neonatal outcomes all healthcare facilities before and after the training intervention

Variable	2018 (n = 7667)	2020 (n = 7265)	p-value	Difference between groups Mean (95% CI)
Apgar score <7 at 5 minutes				
No	7282 (95.0%)	6893 (94.9%)		0.1 (−0.6; 0.8)
Yes	385 (5.0%)	372 (5.1%)	0.81	−0.1 (−0.8; 0.6)
Macerated still deaths				
No	7546 (98.4%)	7150 (98.4%)		0.0 (−0.4; 0.4)
Yes	121 (1.6%)	115 (1.6%)	1.00	−0.0 (−0.4; 0.4)
Fresh stillbirths				
No	7626 (99.5%)	7235 (99.6%)		−0.1 (−0.4; 0.1)
Yes	41 (0.5%)	30 (0.4%)	0.34	0.1 (−0.1; 0.4)
Neonatal deaths (0–28 days after birth)				
No	7582 (98.9%)	7171 (98.7%)		0.2 (−0.2; 0.5)
Yes	85 (1.1%)	94 (1.3%)	0.33	−0.2 (−0.5; 0.2)

For categorical variables n (%) is presented. For comparison between groups Fisher´s Exact test (lowest 1-sided p-value multiplied by 2) was used for dichotomous variables. The

Confidence interval for dichotomous variables is the unconditional exact

Confidence limits. If no exact limits can be computed the asymptotic Wald

Confidence limits with continuity correction are calculated instead

There were less emergency caesarean sections (13.0% vs 15.3%) and more vaginal birth (84.7% vs 87.0%) after versus before the training intervention (p = 0.0001). There was a slight increase in giving prophylactic uterotonic after vaginal birth although it was high even before (99.9% vs 99.4%, p = 0.0001). Complications in terms of postpartum blood loss > 500 ml, retained placenta > 30 minutes, Apgar score < 7 at 5 minutes did not differ. Maternal and neonatal deaths did not differ significantly before vs after the training.

## Discussion

To our knowledge, this is the first report on a training programme in a low-income context aiming at establishing holistic person-centred evidence-based childbirth care routines that promote normal physiological birth, prevent and manage complications during labour and birth, and strengthen healthcare professionals’ self-reflection skills and self-confidence. This study shows that the three-pillar training programme was successful. It was delivered as intended, so fidelity, dose, adaptation, and reach were high. Several improved care routines were established. These include improved planning, teamwork, and self-reflection skills in combination with improved awareness of the care environment, of having a respectful encounter, using alternative techniques, and of involve the birthing woman’s companion. The statistics for the year after the training show that mode of birth changed, with fewer emergency caesarean sections and more vaginal births. There was no difference in maternal and neonatal mortality, which was an expected result as these variables are rare in the total number of births and thus demand a greater numbers of births.

It is well known that achievement of high implementation fidelity is important if an intervention is to be successful [22]. One main likely explanation of why this three-pillar training programme succeeded is that it was well accepted in the implementation context. It was surprisingly useful, with almost no need for alteration during implementation. In combination with conducting the training programme in different levels of healthcare facilities, this means that we can assume that it will be replicable, accepted, and useful elsewhere in urban settings in the DRC and in similar low-income contexts.

Another reason for the success was probably the use of low-dose, high-frequency principles in the training programme [20]. The Helping Mother Survive Bleeding after Birth [9] and the Helping Babies Breath simulation-based training programmes [10] have been proven to improve competence among care providers, resulting in improved maternal and neonatal health outcomes in different low-income settings. Our training programme used both these programs in pillar 2. Our study found that the dose and frequency of 15–60-minute training sessions were acceptable to the healthcare providers. This is in accordance with the findings of a study in Uganda using low-dose, high-frequency training to prevent and treat postpartum haemorrhage and neonatal asphyxia [23].

The unique innovation in our intervention is that, in addition to training on preventing and treating bleeding and on acting immediately after birth, including providing resuscitation (pillar 2), it covered skills required to promote and assist normal physiological births (pillar 1) and to develop self-reflection skills and professional self-confidence (pillar 3).

Conducting process-oriented group reflection following a structured model [19] never used in a low-income setting, was found to be very useful in developing and strengthening professional identity and self-confidence. None of the participants had ever been in such reflection groups, in either basic education, clinical practice, or continuing education. Group reflection clearly developed the participants’ ability to reflect on and share their experienced care situations. Our analysis found that a main reason for this was that the reflection and sharing were conducted in a safe environment where feedback was given in a non-judgmental manner and where nothing said in the groups was repeated outside of them.

Our study also found that the training fostered collegial collaboration and teamwork between the healthcare providers. There was awareness that patient safety could be enhanced through improved and more respectful care routines to manage labour in normal and especially complicated care situations. This echo the findings of studies in Tanzania using the training programme Helping Mothers Survive Bleeding after Birth, which also found that skills training in multi-disciplinary teams enhanced teamwork, having a positive effect on the provided care [24,25].

It is remarkable and encouraging that the knowledge obtained in this three-pillar training programme was immediately applied in practice. There was no failure to translate evidence-based knowledge into practice, as earlier identified [11]. Two key factors that we believe contributed to this were the frequently recurring training sessions according to the low-dose, high-frequency philosophy, and that the health facilities were provided with materials that facilitated both the training and implementation in care practice.

A major challenge in implementing the three-pillar training was the lack of incentives given to healthcare providers for participation during their spare time. That lack of compensation is a barrier to participation in in-service training is consistent with findings regarding a training activity for midwives in Uganda [26]. If the three-pillar training programme is to be scaled up, the contextual factors will need to be considered. As found here and in our previous study of contextual factors influencing the implementation of this three-pillar training programme, incentives of various types are crucial contextual factors that influence the training intervention, and therefore must be considered in the planning phase of the intervention.

### Methodological considerations

It is important to research quality-improvement projects [11], and we found using a framework [12] when evaluating the implementation of this training intervention to be very useful. It structured the analysis and helped identify factors that probably would not have been identified during an inductive content analysis.

Anyhow, this study has certain limitations. It was a very small in scale, as it included only selected healthcare facilities from only one health zone, which weakens the generalizability of its findings. Another limitation is that the data collected did not cover everything we wanted to explore, including variables capturing the effects of the training, and salutogenic data such as no use of use of oxytocin to augment labour, skin-to-skin contact, breast-feeding, zero separation between mother and neonate, and the mother’s childbirth experience. Ideally designed studies would incorporate such salutogenic outcomes [27]. This was because it would increase already heavy workloads and would have required additional financial resources. Therefore, we only had access to routinely data reported to the health zone. The study design, in which statistics were collected on group and not on individual level, is another weakness. Thus, it is not possible to conclude that the measured maternal and neonatal changes is an effect of only this training intervention. To show this would have required a prospective controlled study. However, although the study could not confirm a causal relationship, it is highly likely that this training intervention had a substantial contribution in establishing new evidence based care routines that in the long turn can improve maternal and neonatal health.

## Conclusion

This process evaluation provides evidence of the successful implementation of a three-pillar training intervention to promote holistic person-centred, evidence-based childbirth care routines that promote normal physiological births, prevent and manage complications during labour and birth, and strengthen healthcare professionals’ self-reflection skills and self-confidence in the DRC. The training intervention led to several improved care routines, and there were fewer emergency caesarean sections and more vaginal births the year after the intervention was completed. Given these positive results, we highly suggest to use the design of this training intervention programme in DRC and settings with similar conditions. While doing this, we recommend to consider contextual factors such as participation incentives of various types in the planning phase of the intervention, measure salutogenic outcomes, and ensure that re-fresher training is in built in the training programme.

## Data Availability

The datasets used and/or analysed in this study are available from the corresponding author on reasonable request.

## Paper context

As part of a quality-improvement programme in eastern DRC, intended to reduce maternal and neonatal mortality and morbidity, we explored the implementation of a three-pillar training intervention, guided by a framework for process evaluation. The study shows that using a low-dose, high-frequency approach and providing healthcare facilities with necessary equipment can improve care routines. The year after there were fewer emergency caesarean sections and more vaginal births.
